# Generation of Knockout and Transgenic Zebrafish to Characterize Abcc4 Functions in Detoxification and Efflux of Lead

**DOI:** 10.3390/ijms22042054

**Published:** 2021-02-19

**Authors:** Xing Lu, Yong Long, Xixi Li, Lang Zhang, Qing Li, Hua Wen, Shan Zhong, Zongbin Cui

**Affiliations:** 1 Key Laboratory of Freshwater Biodiversity Conservation, Ministry of Agriculture and Rural Affairs, Yangtze River Fisheries Research Institute, Chinese Academy of Fishery Sciences, Wuhan 430223, China; luxing@yfi.ac.cn (X.L.); wenhua.hb@163.com (H.W.); 2 Department of Genetics, Wuhan University, Wuhan 430071, China; lixixi@ihb.ac.cn (X.L.); zhanglang@yfi.ac.cn (L.Z.); 3 State Key Laboratory of Freshwater Ecology and Biotechnology, Institute of Hydrobiology, Chinese Academy of Sciences, Wuhan 430072, China; longyong@ihb.ac.cn (Y.L.); qli@ihb.ac.cn (Q.L.); 4 Guangdong Provincial Key Laboratory of Microbial Culture Collection and Application, State Key Laboratory of Applied Microbiology Southern China, Guangdong Institute of Microbiology, Guangdong Academy of Sciences, Guangzhou 510070, China; 5 Hubei Provincial Key Laboratory of Allergy and Immunology, Wuhan 430071, China

**Keywords:** zebrafish, Abcc4, lead, detoxification, efflux transporter, transgenesis, knockout

## Abstract

Lead (Pb) is one of the major heavy metals that are toxic to vertebrates and usually considered as environmental pollutants. ABCC4/MRP4 is an organic anion transporter that mediates cellular efflux of a wide range of exogenous and endogenous compounds such as cyclic nucleotides and anti-cancer drugs; however, it remains unclear whether ABCC4 and its orthologs function in the detoxification and excretion of toxic lead. In this study, we found that the transcriptional and translational expression of zebrafish *abcc4* was significantly induced under lead exposure in developing zebrafish embryos and adult tissues. Overexpression of zebrafish Abcc4 markedly decreased the cytotoxicity and accumulation of lead in pig renal proximal tubule cell line (LLC-PK1 cells). To further understand the functions of zebrafish Abcc4 in lead detoxification, the clustered regularly interspaced palindromic repeats (CRISPR)/Cas9 system was used to create an *abcc4^−/−^* mutant zebrafish line. In comparison with the wild-type (WT) zebrafish, the *abcc4*^−/−^ mutants showed a higher death rate and lead accumulation upon exposure to lead. Furthermore, a stable *abcc4*-transgenic zebrafish line was successfully generated, which exerted stronger ability to detoxify and excrete lead than WT zebrafish. These findings indicate that zebrafish Abcc4 plays a crucial role in lead detoxification and cellular efflux and could be used as a potential biomarker to monitor lead contamination in a water environment.

## 1. Introduction

Multidrug resistance-associated proteins (MRPs) are ATP-binding cassette (ABC) transporters, which belong to the subfamily C of the ABC superfamily (ABCC) and are capable of transporting a wide range of substrates in ATP-dependent manner [1]. The denomination of MRPs derives from their clinical functions in multidrug resistance, as high-level expression of MRPs in tumor cells often results in lower cellular drug accumulation and stands as a major obstacle to the therapy of disseminated cancers [2]. Besides, MRPs also exert important roles in tissue defense and can protect vital body structures, such as the brain, cerebrospinal fluid, testis and fetus, against the action of toxins [3]. So far, at least nine MRP transporters have been characterized [4]. Most of them (MRP1–8) are reported to be organic anion transporters, and each MRP has its unique membrane localization, tissue distribution and substrate specificity [1,5,6].

ABCC4/MRP4 is the fourth member characterized in the MRP family, which possesses considerable similarities in substrate specificity and structure characteristics to ABCC5/MRP5 [7]. ABCC4 is ubiquitously expressed, with its localization on either the apical membrane of renal proximal tubule cells [8] or the basolateral membrane of hepatocytes [9] and prostate tubuloacinar cells [10]. It has been demonstrated that ABCC4 acts as a versatile efflux transporter to pump a range of structurally diverse exogenous and endogenous compounds out of the cell [6]. First, ABCC4 can mediate the efflux of cyclic nucleotides, nucleoside analogs, eicosanoids, bile acid and steroid conjugates [11]. Second, ABCC4 serves as a drug transporter for the excretion of a wide variety of antiviral, cytostatic, antibiotic and cardiovascular drugs [5,6]. Third, ABCC4 is capable of transporting a broad array of toxicants such as organochlorine pesticides [12,13]. Furthermore, ABCC4 has been shown to exhibit essential functions in tissue defense [14,15].

Lead (Pb) is one of the hazardous heavy metals that are toxic to vertebrates. In addition to the natural weathering processes, lead pollution often originates from mining and smelting activities, lead-containing paints, gasoline and explosives, as well as from the disposal of municipal sewage sludges enriched in lead [16]. Although many control measures have been adopted in many countries to limit lead emissions in the environment, it remains one of the most serious global environmental toxicants [17]. The toxicological effects of lead on human and animal models have been well-characterized in numerous studies [18,19,20]. For instance, high levels of lead accumulation can damage nearly all organ systems, most importantly the central nervous system, kidneys and blood, culminating in death at excessive levels, while low level of lead exposure can impair psychological and neurobehavioral functions, heme synthesis and other biochemical processes.

From natural and anthropogenic processes, lead is also regarded as an important contamination source in a water environment. Once released into water, lead can be absorbed and accumulated by aquatic organisms. Therefore, to avoid the detrimental effects of this nonessential toxic metal, organisms or cells need to develop efficient detoxification and efflux systems. Previous studies have demonstrated that heavy metals such as cadmium (Cd) and mercury (Hg) are able to conjugate with glutathione and finally be pumped out of the cells by some of the MRP transporters [21,22,23]. ABCC4/MRP4, as a major factor of the multi-xenobiotic resistance (MXR) mechanism in aquatic organisms, can recognize many kinds of toxicants conjugating with glutathione, glucuronate or sulfate as substrates [12]. However, it continues to be explained whether ABCC4 functions in the detoxification and efflux of toxic lead.

Zebrafish has been widely used as a vertebrate model for research into embryonic development, genetic analysis, toxicology and various human diseases [24,25]. In this study, we aimed to dissect the roles of zebrafish Abcc4 in the detoxification of lead through the creation of *abcc4*-knockout and transgenic zebrafish lines.

## 2. Results

### 2.1. Induction of Zebrafish abcc4/Abcc4 by Lead Exposure

**Figure 1:**
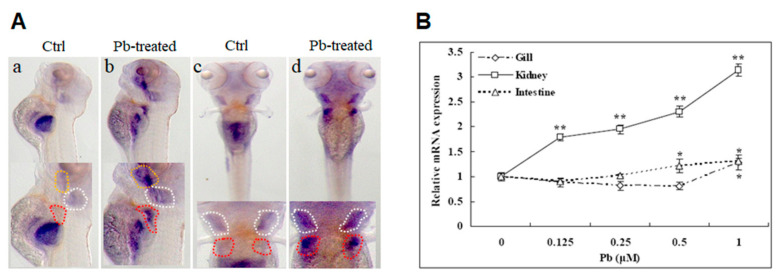
Lead (Pb)-induced expression of the *abcc4* gene in developing embryos and adult tissues of zebrafish. (**A**) Induced expression of the zebrafish *abcc4* gene in developing embryos under lead stress. Embryos were treated with 5 μM lead from 24 to 120 h post-fertilization (hpf) and subjected to whole-mount RNA in situ hybridization (WISH) with antisense RNA probe. (a,b) Lateral views; (c,d) dorsal views. Yellow, white and red dashed lines indicate the oral cavity, gill and pronephric tubules, respectively. (**B**) Transcriptional responses of the zebrafish *abcc4* gene to lead treatments in adult tissues. Adult zebrafish were exposed to various concentrations of lead for 24 h. Gills and intestines were taken from four females, and kidneys were taken from four females and four males. The same tissues from different fish were mixed for isolation of total RNA and subjected to qRT-PCR analysis of *abcc4* mRNA levels relative to the control. One-way analysis of variance (ANOVA) followed by Duncan’s post-hoc test was performed, and the symbol above the error bars indicates a significant difference (* *p* < 0.05 and ** *p* < 0.01) of mRNA levels in the same tissue among the indicated doses of treatments.

The spatiotemporal expression of the *abcc4* gene during embryogenesis in zebrafish was detected with whole-mount RNA in situ hybridization (WISH) in our previous study [12]. The transcriptional responses of zebrafish *abcc4* to lead treatments were examined here in developing embryos. As shown in Figure 1A, in comparison with larvae cultured in a lead-free medium (Ctrl), exposure to 5 μM lead markedly induced *abcc4* expression in the oral cavity (yellow dashed line), gills (white dashed line) and pronephric tubules (red dashed line) at 120 h post-fertilization (hpf). The mRNA/protein levels of *abcc4*/Abcc4 were also determined in adult zebrafish. As shown in Figure 1B, the expression of *abcc4* transcripts in the intestine and kidney was induced by lead exposure in a dose-dependent manner, with the highest induction detected in the kidneys. Moreover, a significantly induced expression of the *abcc4* gene was also found in the gills after exposure to 1 μM lead. Regarding the protein levels, Abcc4 expression in the kidneys was upregulated by lead exposure from 0.25 μM to 1 μM (Figure S1A–B), but no significant differences were determined in intestines and gills (Figure S1C–F).

Taken together, these findings indicate that the expression of zebrafish *abcc4*/Abcc4 in developing embryos and adult tissues can be induced by lead exposure.

### 2.2. Zebrafish Abcc4 Functions in the Detoxification and Excretion of Lead in LLC-PK1 Cells

**Figure 2:**
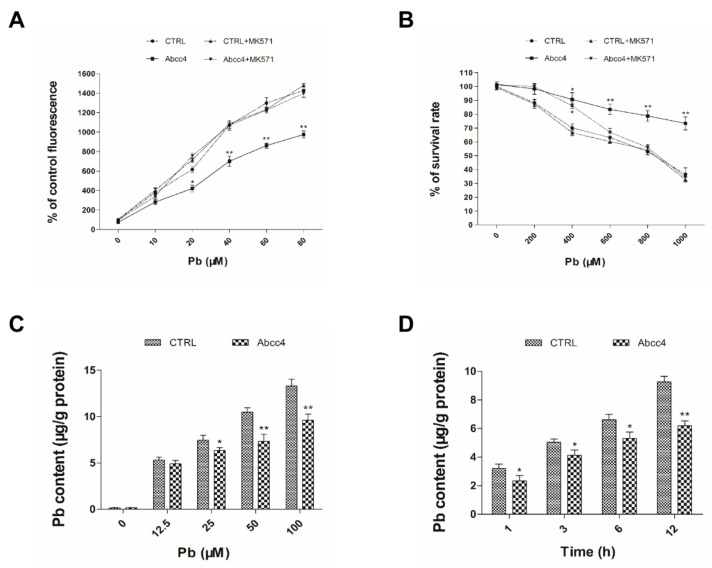
Cellular functions of zebrafish Abcc4 in lead excretion. **(A**) The contents of monochlorobimane (MCB) in LLC-PK1 cells expressing zebrafish Abcc4 and transfected empty vectors (CTRL) with or without 25 μM MK571 were determined with dye accumulation assays after exposure to lead at the indicated concentrations for 24 h. (**B**) Survival rates of LLC-PK1 cells expressing zebrafish Abcc4 and CTRL with or without 25 μM MK571 were determined with 3-[4, 5-dimethylthiazol-2-yl]-2, 5 diphenyl tetrazolium bromide (MTT) assays after exposure to lead at the indicated concentrations for 24 h. (**C**) Contents of lead in LLC-PK1 cells expressing Abcc4 and CTRL after exposure to lead at the indicated concentrations for 12 h. (**D**) Contents of lead in LLC-PK1 cells expressing Abcc4 and CTRL after exposure to 50 μM lead at the indicated time points. Values are expressed as means ± SD (*n* = 3). Significant differences are indicated as * *p* < 0.05 and ** *p* < 0.01.

To gain insights into the functions of zebrafish Abcc4 at the cellular level, dye accumulation assays were performed to detect the effects of lead on the accumulation of monochlorobimane (MCB) in empty vector-transfected (CTRL) and Abcc4-expressing pig renal proximal tubule cell line (LLC-PK1 cells). As shown in Figure 2A, MCB amounts accumulated in CTRL and Abcc4-expressing cells increased markedly with the increase in lead concentrations, and MCB levels of Abcc4-expressing cells were significantly lower than those in CTRL cells after exposure to 20 to 80 μM of lead. However, in the presence of an ABCC-specific inhibitor, MK571, MCB levels in Abcc4-expressing cells (Abcc4 + MK571) were almost the same as those in CTRL cells (CTRL + MK571).

Next, 3-[4, 5-dimethylthiazol-2-yl]-2, 5 diphenyl tetrazolium bromide (MTT) assays were utilized to examine effects of zebrafish Abcc4 on the survival ability of LLC-PK1 cells after exposure to lead. As shown in Figure 2B, the survival rates of zebrafish Abcc4-expressing cells were higher than those of CTLR cells with significant improvement by 1.29- to 2.01-fold after exposure to lead at concentrations of 400–1000 μM. However, when MK571 was added, the survival rates of Abcc4-expressing cells (Abcc4 + MK571) were similar to those of CTRL cells (CTRL + MK571) after treatment with 600–1000 μM lead.

To further assess the effects of zebrafish Abcc4 on the excretion of lead in LLC-PK1 cells, as shown in Figure 2C, lead levels accumulated in Abcc4-expressing cells were significantly lower than those in CTRL cells after treatment with 25–100 μM lead for 12 h. In addition, lead contents in Abcc4-overexpressing cells exposed to 50 μM lead for 1 to 12 h were significantly lower than those in CTRL cells, although an increase of lead accumulation in a time-dependent manner was found in Abcc4-overexpressing cells (Figure 2D).

Overall, these data suggest that zebrafish Abcc4 plays a crucial role in the cellular detoxification and excretion of lead.

### 2.3. Generation of an abcc4-Knockout Zebrafish

**Figure 3:**
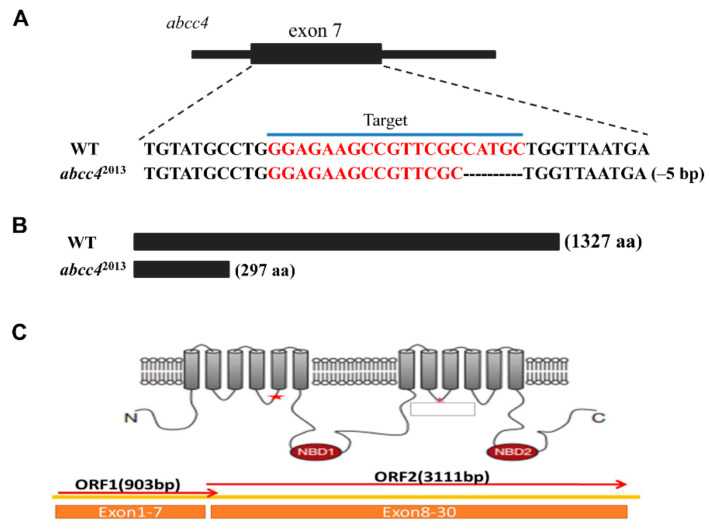
Generation of zebrafish *abcc4*^−/−^ mutants with a clustered regularly interspaced palindromic repeats (CRISPR)/Cas9 system. The red color indicates the sgRNA target sequences for zebrafish *abcc4* gene. (**A**) Diagram of the target site in the zebrafish *abcc4* genome. (**B**) Abcc4 amino acids of wild-type (WT) and *abcc4*^−/−^ mutants. (**C**) The predicted truncation of zebrafish Abcc4 protein. N and C indicate N terminal and C terminal, respectively. The red star symbol indicates a sgRNA targeting site of the *abcc4* gene.

In this study, we designed and generated a sgRNA targeting Exon 7 of the *abcc4* gene (Figure 3A). The clustered regularly interspaced palindromic repeats (CRISPR)-induced mutation was detected by PCR assays. We found the deletion of 5 base pairs (bp) in F_0_ mutants of zebrafish (Figure 3A). This mutation led to a truncated Abcc4 protein containing only 297 amino acids (Figure 3B) and a termination codon at the target site in Exon 7 (Figure 3C).

**Figure 4:**
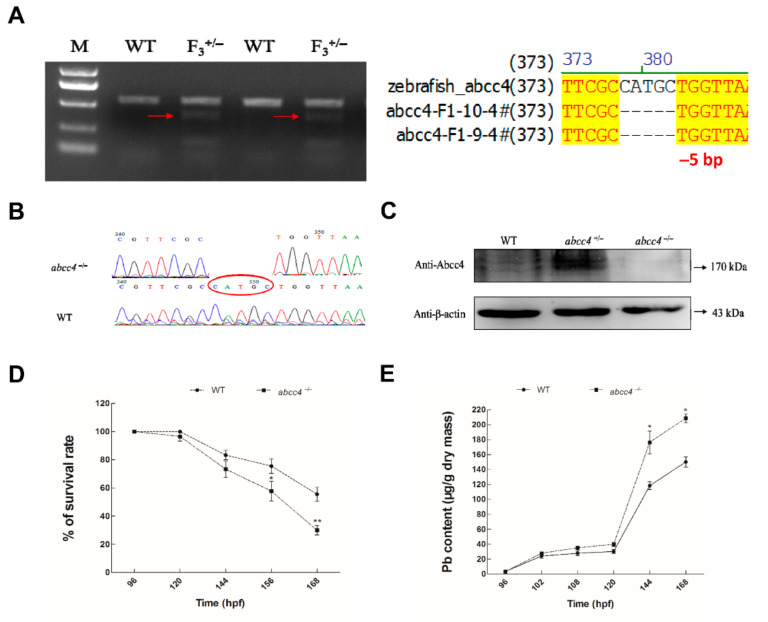
Identification and functional characterization of zebrafish *abcc4*^−/−^ mutants. (**A**) PCR amplification of WT and mutant F_3_ zebrafish. M: Molecular markers; WT: wild type zebrafish; F_3_^+/−^: heterozygous mutants. Red arrows point to the specific DNA bands for mutants. (**B**) Sequencing maps of WT and homozygous mutants. Oval frame: the 5-bp (CATGC) deletion in homozygotes. (**C**) The expression of Abcc4 protein in WT, *abcc4*^+/−^ and *abcc4*^−/−^ larvae at 96 hpf detected with Western blotting. (**D**) Survival rates of *abcc4*^−/−^ and WT larvae were monitored after exposure to 400 μM lead from 96 to 168 hpf. (**E**) Lead contents in *abcc4*^−/−^ and WT larvae after treatment with 10 μM lead at the indicated exposure time points. Values are expressed as means ± SD (*n* = 3). Significant differences are indicated by * *p* < 0.05 and ** *p* < 0.01.

The PCR results from WT and homozygous F_3_ larvae are shown in Figure 4A. Compared with WT larvae (Figure 4B), homozygous larvae (*abcc4^−/−^*) showed a 5-bp deletion (CATGC within the oval frame). Moreover, there were no double peaks in the sequencing maps, which was consistent with the PCR results. Furthermore, the expression of endogenous Abcc4 protein was examined with Western blotting. As shown in Figure 4C, no Abcc4 expression was found in *abcc4^−/−^* larvae, which can survive to adulthood and are fertile under normal rearing conditions. Thus, we successfully generated an *abcc4*^−/−^ mutant zebrafish line using the CRISPR/Cas9 system.

To elucidate the function of endogenous Abcc4 in lead detoxification, we assessed the survival rate of wild-type (WT) and *abcc4*^−/−^ larvae after lead exposure. As shown in Figure 4D, after exposure to 400 μM lead from 156 to 168 hpf, the survival rates of *abcc4*^−/−^ larvae were significantly lower than those of WT larvae. We then assessed the effects of lead on the accumulation of WT and *abcc4*^−/−^ larvae. As shown in Figure 4E, lead contents accumulated in *abcc4*^−/−^ larvae from 144 to 168 hpf were significantly higher than those in WT larvae, and increased in a time-dependent manner by an average of 27.80% after exposed to 10 μM lead for 72 h. Thus, knockout of endogenous Abcc4 led to the sensitivity of zebrafish larvae to lead due to an increased accumulation of lead.

### 2.4. Generation of an abcc4-Transgenic Zebrafish Line

**Figure 5:**
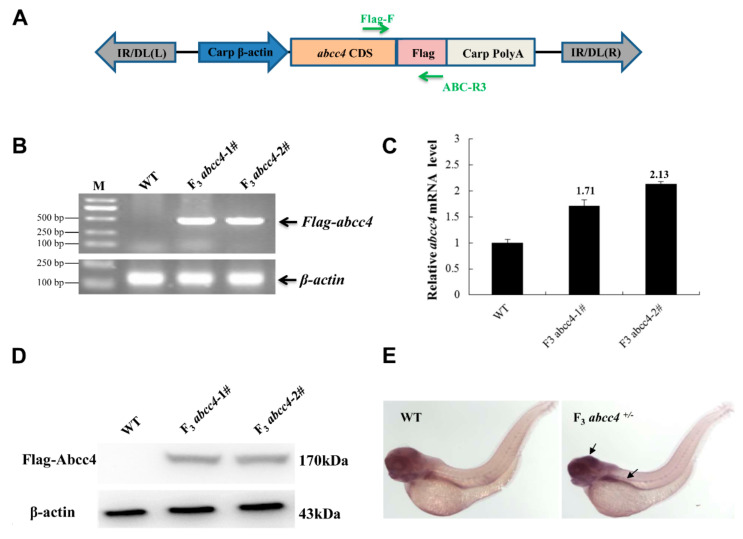
Expression of foreign Abcc4 in transgenic zebrafish. (**A**) A primer pair (Flag-F and ABC-R3) was designed to recognize the *abcc4* coding sequence (CDS) and Flag sequence. (**B**) Positive F_2_ fish were individually crossed with WT fish to obtain positive F_3_ embryos. Sixty larvae at 96 hpf were pooled for RNA extraction and subsequent RT-PCR analysis with a primer pair (Flag-F and ABC-R3). WT: Wild-type zebrafish embryos at the same developmental stage. F_3_ *abcc4*-1# and F_3_ *abcc4*-2# indicated the pooled larval zebrafish at 96 hpf from two positive F_2_ zebrafish, which were individually crossed with WT fish, respectively. (**C**) qPCR analysis for *abcc4* mRNA levels with a primer pair (*abcc4*–qPCR-F and *abcc4*–qPCR-R). (**D**) Western blotting analysis of Flag-tagged Abcc4 expression in F_3_ embryos. (**E**) WISH analysis of *abcc4* expression in WT and transgenic zebrafish larvae. Arrow heads indicate the brain and intestine.

To further dissect the role of elevated Abcc4 expression in lead detoxification, an *abcc4*-transgenic zebrafish line was generated. As shown in Figure 5A, a plasmid of pT_2_/β-actin P-Abcc4-Flag-β-actin-pA was constructed for the transgenesis, which contains a strong promoter and a polyadenylation signal sequence from the carp *β-actin* gene, and the zebrafish *abcc4* coding sequence (CDS) tagged by a Flag at the carboxyl terminus for transgene expression analysis. The constructed plasmid was co-injected with capped SB11 mRNA into one-cell-stage embryos. These embryos were reared to adulthood and the genomic DNA samples were isolated from their tails for positive screening of foreign *abcc4* cDNA. Three PCR primers were designed against the carp β-actin promoter, the *abcc4* coding sequence and a Flag-tag (Figure S2A). The specificity of the three primer sets was examined by amplification of WT genomic DNA containing certain copies of transgenic plasmids. As shown in Figure S2B, all of these primer sets were suitable for screening positive *abcc4*-transgenic zebrafish.

The positive rate of F_0_ transgenic fish was about 22.0% (Figure S3A–B and Table 1). These positive F_0_ fish were individually mated with WT fish to produce offspring. PCR analysis indicated that only 3 of 79 F_0_ positive individuals successfully transmitted the foreign *abcc4* cDNA to the F_0_ offspring (data not shown), probably due to a mosaic distribution of the *abcc4*-transgene in cells or tissues of the F_0_ fish. F_1_ embryos were reared to adulthood and 47.73% of the F_1_ fish carried the *abcc4*-transgene in the genome (Table 1). In addition, the F_2_ offspring were obtained by intercrossing positive F_1_ fish, and 36.11% of F_2_ fish carried the *abcc4*-transgene in the genome (Table 2). These findings indicate that we have successfully generated a transgenic zebrafish line with stable transmission of the *abcc4*-transgene.

To examine the expression of a target gene tagged with the Flag sequence in transgenic zebrafish, F_3_ embryos were obtained by outcrossing *abcc4*-positive F_2_ fish with WT fish. As shown in Figure 5B, RT-PCR assays showed that the Flag-*abcc4* cDNA can be detected at the transcriptional level using the primer set Flag-F and ABC-R3 (Table 2).

The qPCR results indicated that the transcriptional level of the *abcc4* gene in transgenic zebrafish was improved by 1.71–2.13-fold when compared with that of WT (Figure 5C). Furthermore, Western blotting analysis demonstrated that zebrafish Abcc4 proteins are properly expressed under the control of the carp β-actin promoter in F_3_ embryos (Figure 5D). WISH showed that in comparison with the WT, elevated levels of foreign *abcc4* expression were mainly detected in the brain and intestine of transgenic embryos at 96 h post-fertilization (hpf) (Figure 5E). These data suggested that the *abcc4*-transgenic zebrafish line was suitable for the subsequent analysis of Abcc4 functions.

### 2.5. Functional Characterization of the abcc4-Transgenic Zebrafish

**Figure 6:**
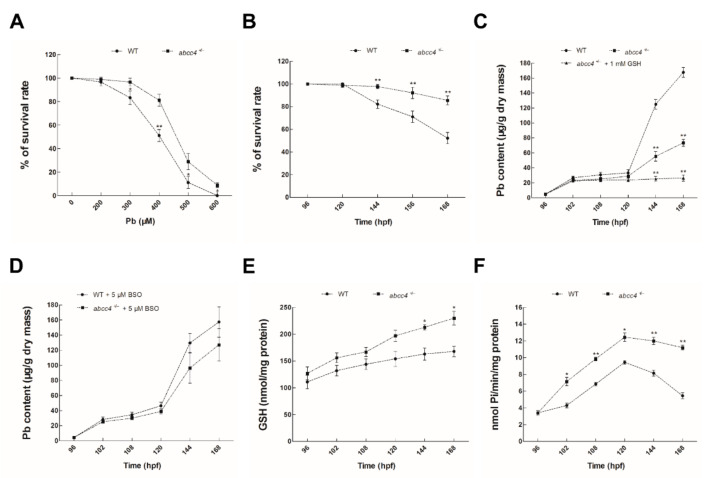
Functional characterization of Abcc4 in lead detoxification in transgenic zebrafish. (**A**) Survival rates of *abcc4*-transgenic and WT embryos were monitored after treatment with different concentrations of lead (0–600 μM) from 96 to 168 hpf. (**B**) Survival rates of *abcc4*-transgenic and WT larvae were detected after exposure to 400 μM lead for 72 h. (**C**) Contents of lead in *abcc4*-transgenic and WT larvae after exposure to 10 μM lead with or without 1 mM glutathione (GSH) at the indicated exposure time points. (**D**) Contents of lead in *abcc4*-transgenic and WT larvae at the indicated exposure time points after treatment with a medium containing 10 μM lead and simultaneously with 5 μM buthionine sulfoximine (BSO), an inhibitor of GSH biosynthesis. (**E**) Intracellular GSH contents in *abcc4*-transgenic and WT larvae exposed to 10 μM lead at the indicated exposure time points. (**F**) ATPase activities as shown by inorganic phosphate (Pi) levels in *abcc4*-transgenic and WT larvae after treatment with 10 μM lead at the indicated exposure time points. Values are expressed as means ± SD (*n* = 3). Significant differences are indicated by * *p* < 0.05 and ** *p* < 0.01.

First, we examined the ability of *abcc4*-transgenic and WT zebrafish larvae for detoxification after treatment with lead from 96 to 168 hpf. As shown in Figure 6A, the survival rates of *abcc4*-transgenic larvae at 168 hpf were significantly higher than those of WT larvae after exposure to 300–600 μM lead (*p* < 0.05 or *p* < 0.01). Moreover, when exposed to 400 μM lead from 96 to 168 hpf, the survival rates of *abcc4*-transgenic larvae from 144 to 168 hpf were significantly higher than those of WT larvae (*p* < 0.01) (Figure 6B).

Next, we investigated the ability of *abcc4*-transgenic and WT zebrafish larvae to excrete lead. As shown in Figure 6C, the lead contents accumulated in *abcc4*-transgenic zebrafish larvae were significantly lower than those in WT larvae and dropped by an average of 43.68% in a time-dependent manner after treatment with 10 μM lead for 72 h. Moreover, when 1 mM glutathione (GSH) was added, the contents of lead in larvae (Abcc4^+/−^ + 1 mM GSH) exposed to 10 μM lead were markedly lower than those in WT (with an average decrease of 64.76%) or *abcc4*-transgenic larvae (Abcc4^+/−^, with an average decrease of 37.43%) (Figure 6C). However, no significant difference was found in the accumulation of lead between WT and *abcc4*-transgenic larvae after co-treatment with 10 μM lead and 5 μM buthionine sulfoximine (BSO), an inhibitor of GSH biosynthesis (Figure 6D).

It was reported that excretion of heavy metals by MRPs/ABCCs requires the coordination of reduced glutathione (GSH) [26], so we analyzed GSH levels in developing embryos to investigate the mechanisms underlying lead detoxification. As shown in Figure 6E, treatments of WT or *abcc4*-transgenic larvae with 10 μM lead markedly increased GSH levels in a time-dependent manner, and the contents of GSH in *abcc4*-transgenic larvae were significantly higher than those in WT larvae after treatment with 10 μM lead from 144 to 168 hpf. Furthermore, levels of inorganic phosphate (Pi) were detected to monitor the hydrolysis of ATP in developing embryos exposed to lead. As shown in Figure 6F, Pi levels in *abcc4*-transgenic larvae exposed to 10 μM lead from 102 to 168 hpf were higher than those in WT larvae and increased in a time-dependent manner by an average of 32.92%. These data indicate that the detoxification and excretion of lead by zebrafish Abcc4 require the involvement of GSH and the hydrolysis of ATP.

## 3. Discussion

Lead is widely distributed in the water environment and can gain entry into different tissues and cells of fish through contaminated water and the aquatic food chain. Upon entering cells, this nonessential metal exerts multiple adverse effects through interfering with the functions of essential metals such as Zn and Fe [27]; generating reactive oxygen species (ROS) [28]; distributing physiological signal transduction [29]; affecting gene expression; inducing damage to DNA, membranes and proteins; and inhibiting DNA repair [30]. To cope with the toxicity of nonessential toxic heavy metals, vertebrates, including fish have evolved detoxification/defense systems. It is suggested that most members of the MRP/ABCC subfamily proteins function as organic anion transporters, which can extrude a variety of substrates, including anti-cancer drugs and glutathione, glucuronide and sulfate conjugates of diverse compounds [31], so they play an important role in cellular protection against endo- and exogenous toxic compounds. MRP1/ABCC1 and MRP2/ABCC2 are the best characterized and toxicologically relevant MRP/ABCC proteins in the livers of mammals, which are expressed in the basolateral and apical membranes of hepatocytes, respectively [3]. Our previous studies have revealed that zebrafish Abcc1 [32], Abcc2 [33] and Abcc5 [34] play vital roles in the efficient detoxification and efflux of heavy metals. MRP4/ABCC4 is a versatile efflux transporter for a wide range of substrates with broad specificity and complex interactions, including endogenous molecules such as cyclic adenosine monophosphate (cAMP) and cyclic guanosine monophosphate (cGMP), physiological metabolites and many kinds of drugs [6]. However, it remains largely unknown whether Abcc4 can function in the detoxification of heavy metals such as lead. In this study, we have elucidated the roles of zebrafish Abcc4 as an efflux transporter of lead in LLC-PK1 cells, and knockout and transgenic zebrafish.

Several lines of evidence from this study suggest that zebrafish Abcc4 plays an important role in lead detoxification through cellular elimination of lead. (i) In developing embryos, lead exposure induced *abcc4* expression in the oral cavity and gills at 120 hpf. The oral cavity and gills of aquatic organisms constitute the main interface between the organism and its environment, and are among the major excretion and detoxification systems in fish [35]. ABC transporters were demonstrated to form an active and physiological barrier at the tissue–environment interface in mussel gills, providing protection against environmental xenotoxicants [36]. Thus, the relatively high-level expression of Abcc4 in zebrafish gills and oral cavities suggests that Abcc4 may play an important role in limiting the uptake or excretion of toxic metal. (ii) In adult zebrafish, exposure to lead induced the transcriptional and protein expression of *abcc4*/Abcc4 in some excretory organs such as kidneys and intestine, indicating that zebrafish Abcc4 is involved in the physiological functions of these organs. (iii) Zebrafish Abcc4 expressed in LLC-PK1 cells exhibited a strong activity in efflux of MCB and lead, and an ABCC-specific inhibitor (MK571) [12] can abolish the efflux activity of zebrafish Abcc4. (iv) When overexpressed in developing embryos, zebrafish Abcc4 functioned in the detoxification and efflux of lead, as shown by the acute toxicity assays and atomic absorption spectrometry. Therefore, zebrafish Abcc4, like its mammalian counterparts, plays a crucial role in the detoxification of various toxicants and is likely involved in tissue defense.

The CRISPR/Cas9 system was used to knockout the endogenous *abcc4* gene in a zebrafish model. We designed a sgRNA targeting Exon 7 and determined its activity. In the F_2_ genotyping, we identified a mutation of a 5-bp deletion in the *abcc4* gene. The 5-bp deletion in Exon 7 led to a frameshift mutation in *abcc4*, resulting in a truncated Abcc4 protein, which caused the loss of the conservative function domain (nucleotide-binding domains (NBDs) and transmembrane domains (TMDs)). We developed an anti-Abcc4 antibody and no Abcc4 expression was detected in the *abcc4*^−/−^ mutant zebrafish. Similar to *abcc4*^−/−^ knockout mice [37], *abcc4*^−/−^ knockout zebrafish are viable and fertile, and have no morphological abnormalities (data not shown), suggesting that Abcc4 does not play an essential role in the development and growth of vertebrates. However, *abcc4*^−/−^ zebrafish mutants exhibited higher death rates and lead accumulation than WT zebrafish after treatment with toxic lead.

The first batch of transgenic fish was generated three decades ago through microinjection of plasmids into fertilized eggs [38]. Since then, many commercial fish species such as carp, tilapia and salmonids have been successfully used for transgenesis [39,40,41]. In addition to commercial transgenic fish, transgenic models of zebrafish and medaka have been developed in many laboratories in order to understand the mechanisms of growth, embryonic development, disease resistance and aspects of certain human diseases [42,43]. In this study, we generated an *abcc4*-transgenic zebrafish line suitable for investigating lead detoxification. The transgenic vector was constructed from the “all-fish” elements, including the zebrafish Abcc4 cDNA and carp β-actin gene promote [44]. We detected the expression of foreign Flag-tagged Abcc4 cDNA in the F_3_ offspring of transgenic zebrafish, indicating that the Abcc4-expressing cassette has stably integrated into the genome. Moreover, the early embryonic development, morphological phenotypes and hatching behavior of the transgenic zebrafish and their ability to produce fertile offspring were not affected by the integration and expression of the *abcc4*-expressing cassette (data not shown). Therefore, this *abcc4*-transgenic zebrafish line provided a valuable resource for the investigation of Abcc4 functions.

ABCC4, as an organic anion transporter, can mediate the efflux of a range of endogenous compounds, such as cyclic nucleotides, nucleoside analogs and bile acids [11]. ABCC4 has also been reported to be involved in the detoxification and excretion of organochlorine pesticides [12,13]. In the present study, we have demonstrated that zebrafish Abcc4 is capable of protecting transgenic zebrafish and LLC-PK1 cells against the toxic effects of lead through promoting lead excretion out of cells. Moreover, lead is able to compete with MCB, a well-known substrate of MRP4/ABCC4 protein, and inhibit the cellular efflux of MCB in LLC-PK1 cells. Obviously, zebrafish Abcc4 functions as an important export pump in tissue defense and lead detoxification. It is noted that the protection against toxic metals is associated with GSH efflux from the cell [45,46,47] and an increased sensitivity to heavy metals can occur when cells are depleted of GSH [23]. In agreement with these observations, we found that a GSH-dependent mechanism is involved in Abcc4-mediated transport of toxic lead in zebrafish.

## 4. Materials and Methods

### 4.1. Chemicals

The analytical grade reagent Pb(NO_3_)_2_ was obtained from Sinopharm Chemical Reagent Co., Ltd., Beijing, China. Monochlorobimane (MCB), MTT, Triton X-100 and dimethylsulfoxide (DMSO) were obtained from Sigma-Aldrich (St Louis, MO, USA). All other chemicals were purchased from commercial sources of the highest purity available.

### 4.2. Zebrafish Maintenance and Lead Treatment

Zebrafish of the AB strain were maintained and bred according to standard protocols [48]. Collection of eggs and culture of embryos were performed following our previous methods [33]. Embryos at different developmental stages were determined according to hours post-fertilization (hpf).

Embryos at 24 hpf were exposed to toxic lead for 96 h. A lead-containing embryo medium was prepared by dilution of lead stock solutions with the embryo medium to the desired concentration (5 μM) and changed once every 12 h. At 120 hpf, 30 larvae from each treatment were fixed and subjected to WISH analysis. The toxicity tests for lead treatment of developing zebrafish embryos were independently performed 3 times.

Zebrafish with mean body weights at 0.47 ± 0.08 g for females and 0.46 ± 0.06 g for males were conditioned for 48 h and then selected for acute lead toxicity. Eight fish per aquarium (4 females and 4 males) were exposed to serial dilutions of lead (0–1 μM) for 24 h. During the exposure period, the water temperature was kept at 28 °C and the fish were deprived of food. At the end of experiment, fish were dissected on ice to obtain tissues for subsequent RNA or protein extraction. The toxicity tests for lead treatment of adult zebrafish were independently performed 2 times.

Abcc4-transgenic, Abcc4-knockout and wild-type embryos at 96 hpf were selected from the same batch and randomly divided into 60 embryos per dish, then treated from 96 to 168 hpf in an embryo medium containing 0, 200, 300, 400, 500 or 600 μM of Pb(NO_3_)_2_. Culture solutions were replaced every 12 h and embryos or larvae that showed no heartbeat and no response to touch were regarded as dead. After lead exposure, the survival rates of developing zebrafish at the corresponding stages or lead concentrations were calculated under a stereomicroscope (Carl Zeiss, NY, USA).

### 4.3. Generation of Transgenic Construct

To construct a transgenic vector pT2/carp β-actin P-Abcc4-Flag-carp β-actin pA, 2 rounds of PCR reactions were performed. First, the carp β-actin polyadenylation (polyA) signal was amplified from the vector pT2/β-actin P-GnRH using the PCR primers BstB I-actin A-F and SBR-actin A-R, and inserted into pT2/SV40-Abcc4-Flag to generate pT2/SV40-Abcc4-Flag-carp β-actin pA. Second, the carp β-actin promoter was also obtained from the vector pT2/β-actin P-GnRH using the primers Sph I-actin P-F and Xma I-actin P-R. The PCR product was digested with *Sph* I and *Xma* I and used to replace the SV40 promoter in pT2/SV40-Abcc4-Flag-carp β-actin pA. The final plasmid construction was then confirmed by sequencing. Capped *Sleeping Beauty* (SB11) transposase mRNA was transcribed in vitro from the linearized pSB11RNAX using the mMESSAGE mMACHINE kit from Ambion (Austin, TX, USA). All primers used are listed in Table 2.

### 4.4. Embryo Microinjection and Cell Culture

Microinjection was performed using an electric microinjector (Eppendorf, Hamburg, Germany). Each zebrafish embryo at the 1-cell stage was microinjected with about 3 nL of solution containing 140 ng/μL of capped SB11 mRNA and 20 ng/μL of circular pT2/carp β-actin P-Abcc4-Flag-carp β-actin pA plasmids at the blastoderm–yolk interface, as described previously [49].

LLC-PK1 cells were cultured in M199 medium supplemented with 3% fetal bovine serum (FBS), 100 units/mL penicillin and 100 μg/mL streptomycin at 37 °C under a 5% CO_2_ humidified atmosphere. The empty vector-transfected (CTRL) and zebrafish Abcc4-overexpressing LLC-PK1 cells were obtained as previously described [12].

### 4.5. Knockout of Zebrafish Abcc4 by the CRISPR/Cas9 System

According to the CRISPR/Cas9 system following established methods [50], sgRNA against the *abcc4* gene (NM_001007038) was designed using the Zifit design website (http://zifit.partners.org/). The sgRNA target sequences for the *abcc4* gene were as follows: GGAGAAGCCGTTCGCCATGC. sgRNA was generated with a PCR-amplified template which contained the T7 promoter and gRNA scaffold sequences, and then synthesized in vitro with T7 RNA polymerase (Thermo Scientific, Waltham, MA, USA). The Cas9-capped mRNA was synthesized using the T7 mMESSAGE mMACHINE Kit from Ambion (Austin, TX, USA) and injected together with sgRNA into fertilized wild-type (WT) eggs.

A PCR assay was conducted to identify CRISPR-induced mutation of zebrafish *abcc4*. The screening primers *abcc4*-F9.14/R9.14 (Table 2) were designed around the *abcc4* sgRNA target site in Exon 7. PCR products were sequenced to verify the efficiency of the target site.

The *abcc4*^−/−^ mutant line was generated. Briefly, the tail fins of the F_0_ adult fish were cut and sequenced using a PCR assay to identify the F_0_ founder. F_0_ positive fish were individually mated with WT fish to generate F_1_ progeny. After 3 months of feeding, the DNA of caudal fin in F_1_ fish was extracted, PCR amplified and sequenced to identify positive F_1_ fish. F_1_ heterozygotes were then self-crossed to generate the F_2_ *abcc4*^−/−^ mutant line.

### 4.6. Genomic DNA Extraction and Transgene Detection

Total genomic DNA from adult fish tails was isolated according to our previous study [51]. Three PCR primers were designed according to the coding sequences of the carp β-actin promoter, *abcc4* CDS and Flag tag (Table 2). The sensitivity and specificity of primers were tested by the addition of transgenic plasmids to the wild-type genomic zebrafish DNA, as described in our previous study [51]. The PCR conditions were as below: 94 °C for 5 min, followed by 30 cycles consisting of 94 °C for 30 s, 60 °C for 30 s and 72 °C for 30 s. A final extension step was set at 72 °C for 10 min.

### 4.7. RNA Isolation, Real-Time PCR and Quantitative Real-Time PCR

Total RNA was extracted using TRIZOL reagent (Invitrogen, Carlsbad, CA, USA). RNA samples were digested with RNase-free DNase I (Promega, Madison, WI, USA). The RNA integrity and quality were examined by electrophoresis and spectrophotometry. The cDNAs were transcribed from 2 μg of total RNA using the RevertAid First Strand cDNA Synthesis Kit from Fermentas (Hanover, MD, USA) in a reaction volume of 20 μL.

To detect the transcriptional expression of *abcc4* in F_3_ transgenic zebrafish., positive F_2_ individuals about 3 months old were individually mated with wild-type fish to obtain the F_3_ transgenic fish. Sixty offspring at 96 hpf were randomly selected for total RNA isolation and cDNA synthesis. The primer set Flag-F and ABC-R3 was used to examine the transcription of *abcc4* and Flag. The reaction was conducted in a total reaction volume of 20 μL containing 0.5 μL of synthesized cDNA as a template. The primer set *β-actin*-F/R was used as a control primer to amplify the cDNA of the *β-actin* gene in zebrafish. All primers are listed in Table 2.

The qRT-PCR was conducted with the SYBR Green Real-time PCR Master Mix (BioRad, Hercules, CA, USA) and the CFX Connect Real-time PCR Detection System (Bio-Rad, Hercules, CA, USA). Primers used for *abcc4* (*abcc4*-qPCR-F/R) and *β-actin* (internal reference) were designed with Primer Premier 5.0 software and are listed in Table 2. The amplification was carried out in a volume of 20 μL containing 10 μL of 2× SYBR Green Real-Time PCR Master Mix, 2 pmol of each primer and 5 μL of 10× diluted cDNA samples. The qPCR programs were as follows: 40 cycles of 10 s at 95 °C and 30 s at 60 °C, followed by the melting curve: 26 cycles of 30 s with an increase of 1 °C between each cycle from 70 °C to 95 °C. Data were expressed as the relative expression of the reference gene using 2^−ΔΔCt^ method [52].

### 4.8. Antibody Preparation and Western Blotting

The partial coding sequence (CDS) of the zebrafish *abcc4* gene was amplified and subsequently inserted into the expression vector pGEX-4T-1 to construct pGEX–Abcc4. The expression of the glutathione-S-transferase (GST)-tagged fusion protein was induced from pGEX–Abcc4 using isopropy1-b-D-thiogalactopyranoside (IPTG). pGEX–4T-1 was used as the control vector. To examine Abcc4 expression, all the collected samples were detected by SDS-PAGE gels. To purify the GST-Abcc4, Abcc4 was expressed in *Escherichia coli* (*E. coli*) BL21 and purified by glutathione-sepharose resin. The concentrations of the soluble proteins were determined using the bicinchoninic acid (BCA) method. Finally, anti-Abcc4 antibodies were prepared by injecting the purified Abcc4 fusion protein into rabbits, and then purifying them using Protein-Sepharose CL-4B.

The protein extraction from embryonic cells was performed following our previous protocol [53]. The adult tissues were homogenized with liquid nitrogen and lysed in a radio-immunoprecipitation assay (RIPA) buffer with phenylmethanesulfonyl fluoride (a protease inhibitor; Amresco, OH, USA). The protein concentrations were measured using the BCA method (Beyotime, Nantong, China). Equal amounts of protein (20 μg) were separated by SDS-PAGE and electrotransferred on Immobilon-P Trasfer Membranes from Millipore. Western blotting and enhanced chemiluminescence (ECL) detection were performed according to our previous protocol [54]. Protein signals were detected using Fujifilm LAS-4000, and their densities were calculated by ImageJ. software (National Institutes of Health, Bethesda, MD, USA). An anti-Flag antibody was purchased from Sigma-Aldrich. The measured protein levels of Abcc4 were normalized to that of β-actin.

### 4.9. Dye Accumulation Assay

The pig kidney-derived LLC-PK1 cell has been widely used as an ideal in vitro model for membrane transporter functions due to its monolayer junctions and low levels of endogenous transporters [55,56]. The stable cell lines transfected with zebrafish Abcc4 and the empty vector (CTRL) have been characterized in our previous study [12].

MCB, an excellent fluorescent dye substrate of mammalian ABCC transporters, was used in this experiment to investigate the effects of lead on the accumulation of MCB in the CTRL and Abcc4-expressing LLC-PK1 cells. The method was performed as described previously [12]. The fluorescence was measured using a microplate reader (Spectra-Max M5, Molecular Devices, Sunnyvale, CA, USA) at 390 nm excitation and 480 nm emission wavelengths.

### 4.10. Cytotoxicity Assay

The viability of LLC-PK1 cells were measured with MTT assays after exposure to toxic lead from 0 to 1000 μM. The method was performed as described previously [57]. Absorbance of each well was read at 540 nm with a microplate reader. Viability was expressed as a percentage of the corresponding control. All the experiments were performed at least 3 times.

### 4.11. Whole-Mount RNA In Situ Hybridization

RNA probes were synthesized using the DIG RNA Labeling Kits (T3 or T7) from Roche Applied Science (Indianapolis, IN, USA). Whole-mount RNA in situ hybridization (WISH) was performed as previously described [58]. Images were taken on a stereomicroscope from Zeiss with a color charge coupled device (CCD) camera. The experiment was conducted at least 2 times.

### 4.12. Atomic Absorption Spectrometry Detection

LLC-PK1 cells expressing Abcc4 or CTRL were treated with different concentrations of lead (0–100 μM), then washed with phosphate buffered saline (PBS) and collected at 1, 3, 6 and 12 h after treatment. Abcc4-transgenetic with or without 1 mM GSH or 5 μM BSO, wild-type (WT) with or without 5 μM BSO and Abcc4-knockout zebrafish embryos were exposed to 10 μM lead, washed with the embryo medium and collected at 96, 102, 108, 120, 144 and 168 h after treatment. All samples were lysed in nitric acid at 65 °C for 24 h and then measured using atomic absorption spectrometry (AAS, Varian AA240) to detect lead contents, as described previously [33].

### 4.13. Detection of Glutathione and ATPase Activity

Wild-type and Abcc4-transgenetic developing embryos were exposed to 10 μM lead from 96 to 168 hpf and collected at the indicated stages. GSH levels and ATPase activity were measured by an enzymatic method or inorganic phosphate liberation, as described in our previous protocol [12].

### 4.14. Statistical Analysis

Values were expressed as means ± standard deviation (SD). Student’s test or one-way analysis of variance (ANOVA) followed by Duncan’s post-hoc test was performed using SPSS 18.0 (Inc., Chicago, IL, USA). Differences were considered significant at *p* < 0.05.

## 5. Conclusions

In this study, we successfully generated stable *abcc4*-knockout and transgenic zebrafish lines and demonstrated that zebrafish Abcc4 can act as an efflux transporter of lead in developing zebrafish and LLC-PK1 cells. Considering the wide distribution of toxicants in the water environment and the interaction of Abcc4 with its broad substrates, *abcc4*-knockout and transgenic zebrafish lines can be used for further elucidation of the molecular mechanisms underlying the transport and detoxification of various other toxicants.

## Figures and Tables

**Table 1:** PCR screening of *abcc4*-transgenic zebrafish.

Generation of Transgenic Fish	Positive Fish Number	Total Fish Number	Positive Frequency (%)
F_0_	79	357	22.13
F_1_	63	132	47.73
F_2_	26	72	36.11

**Table 2:** PCR primers used in the study.

Primer Names	Sequences (5′–3′)	Description	Amplicon Size
*BstB I-actin A-F*	GATTTCGAAAAACGGACTGTTACCACTTCACG	Cloning, the *BstB* I site is underlined	982 bp
*SBR-actin A-R*	CCATGATTACGCCAAGCTCG	Cloning	
*Sph I-actin P-F*	AGCTTGCATGCTCAAACTGTGGCACCATCT	Cloning; the *Sph* I site is underlined	2134 bp
*Xma I-actin P-R*	TGTCCCGGGCTGAACTGTAAATGAATGAG	Cloning; the *Xma* I site is underlined	
*Carp actin P-F*	CAGGAATGCAAGCCTGATTC	Transgene detection	460 bp
*Carp actin P-R*	GAAGCTGGATTGTTTGAAGAGC	Transgene detection	
*abcc4-F*	CTTCAGGACTGGTGGCTTTC	Transgene detection	468 bp
*abcc4-R*	CAGGAACAGGAAGCAAATCAAC	Transgene detection	
*Flag-F*	GTCAACGGCAGCTCGTCTGTCTG	Transgene detection	440 bp
*ABC-R3*	GTCATCGTCGTCCTTGTAGTC	Transgene detection	
*abcc4-qPCR-F*	GTCCGCCTCACCGTCACTC	qPCR	150 bp
*abcc4-qPCR-R*	CGGCTCTTTCTTCTCCTCCTG	qPCR	
*abcc4-F9.14*	CCTTCCCAATACCATAATCACACT	Knockout mutant detection	
*abcc4-R9.14*	CAAGGTTGAGACTTGAAGCACAG	Knockout mutant detection	
*β-actin-F*	CGAGCAGGAGATGGGAACC	qPCR	102 bp
*β-actin-R*	CAACGGAAACGCTCATTGC	qPCR	

Note: TTCGAA indicates the *BstB* I site; GCATGC indicates the *Sph* I site; CCCGGG indicates *Xma* I site.

## Data Availability

The data presented in this study are available in the article and supplementary material.

## Supplementary Materials

The following are available online at https://www.mdpi.com/1422-0067/22/4/2054/s1, Figure S1: Pb induced expression of Abcc4 protein in tissues of adult zebrafish, Figure S2: The design and optimization of PCR primers, Figure S3: Screening *abcc4*-transgenic zebrafish in F_1_ generation.

## Abbreviations

ABC	ATP-binding cassette transporters
ABCC	Subfamily C of ABC superfamily
ANOVA	One-way analysis of variance
ATP	Adenosine triphosphate
BCA	Bicinchoninic acid
BSO	Buthionine sulfoximine
bp	Base pair
cAMP	cyclic adenosine monophosphate
CCD	Charge coupled device
Cd	Cadmium
CDS	Coding sequence
cGMP	cyclic guanosine monophosphate
CRISPR	Clustered regularly interspaced palindromic repeats
CTRL	Empty vector-transfected
DMSO	Dimethyl sulfoxide
DOAJ	Directory of open access journals
ECL	Enhanced chemiluminescence
*E. coli*	*Escherichia coli*
GSH	Glutathione
GST	Glutathione-S-transferase
Hg	Mercury
Hpf	Hours post-fertilization
IPTG	Isopropy1-b-D-thiogalactopyranoside
LLC-PK1	Pig renal proximal tubule cell line
MCB	Monochlorobimane
MDPI	Multidisciplinary Digital Publishing Institute
MRP	Multidrug resistance-associated protein
MTT	3-[4, 5-dimethylthiazol-2-yl]-2, 5 diphenyl tetrazolium bromide
MXR	Multi-xenobiotic resistance
NBDs	Nucleotide-binding domains
Pb	Lead
PBS	Phosphate buffered saline
PCR	Polymerase chain reaction
qPCR	Quantitative real-time PCR
RIPA	Radio-immunoprecipitation assay
RT-PCR	Real-time PCR
ROS	Reactive oxygen species
SD	Standard deviation
TMDs	Transmembrane domains
WISH	Whole-mount RNA in situ hybridization
WT	Wild-type
