# Supplementary material for: Generation of Knockout and Transgenic Zebrafish to Characterize Abcc4 Functions in Detoxification and Efflux of Lead
Source: Int J Mol Sci. 2021 Feb 19;22(4):2054. doi: 10.3390/ijms22042054 (PMC7923114; doi:10.3390/ijms22042054)
Supplement: Supplementary file 1 [file ijms-22-02054-s001.pdf]

Supplemental Figures and legends:

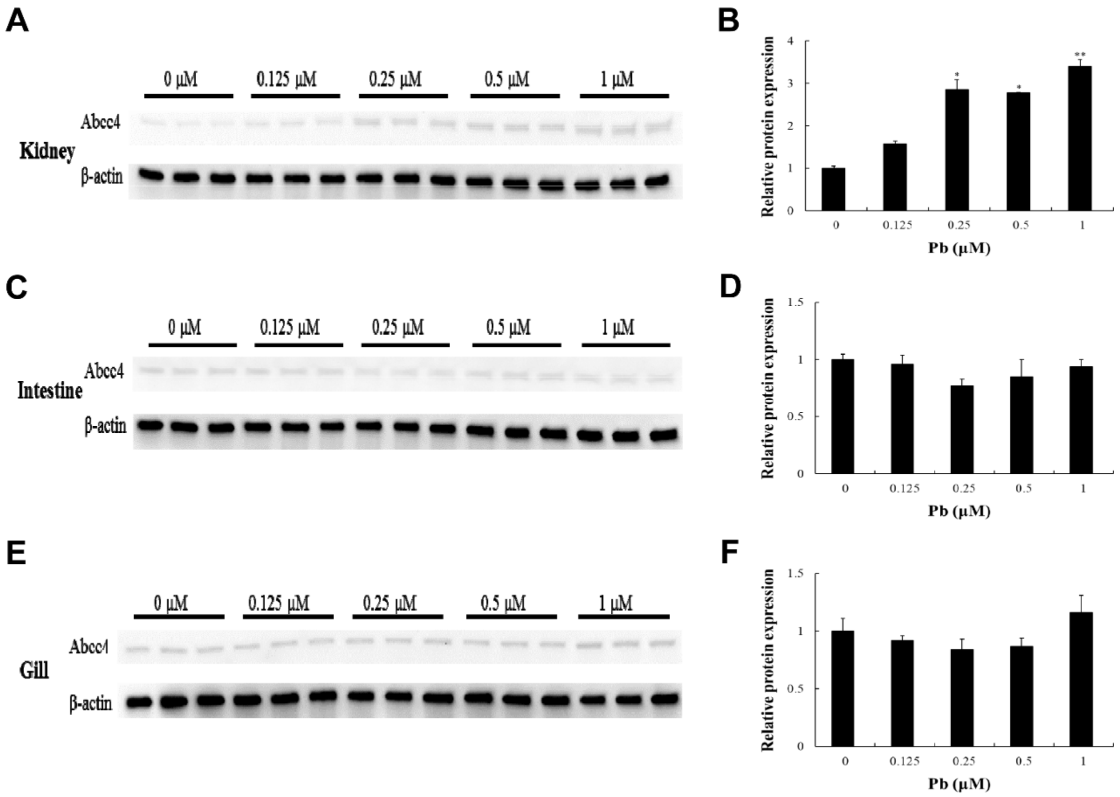

Figure S1- Pb induced expression of Abcc4 protein in tissues of adult zebrafish. Adult zebrafish were exposed to various concentrations of lead for 24 hours. Gills and intestines were taken from 4 females, and kidneys were taken from 4 females and 4 males. Same tissues from different fish were mixed for isolation of total proteins and subjected to western blotting analysis of Abcc4 protein levels relative to the control. The β-actin was used as a loading control. One-way analysis of variance (ANOVA) followed by Duncan's post-hoc test was performed and the symbol above error bars indicate a significant difference (*$p < 0.05$ and **$p < 0.01$) of protein levels in the same tissue among distinct doses of treatments.

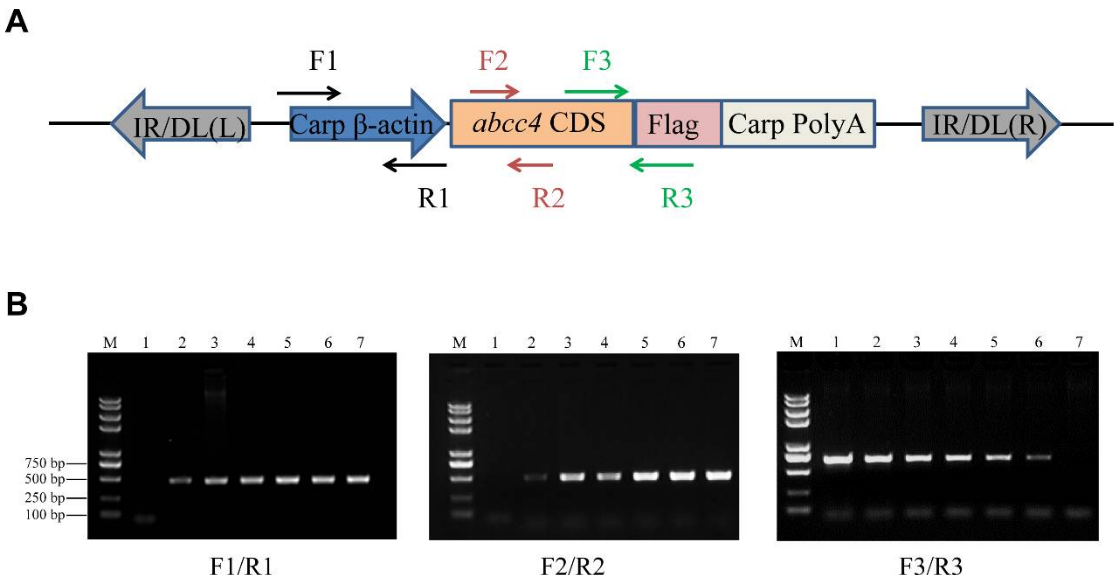

Figure S2-The design and optimization of PCR primers.

(A) Primers designed on Abcc4-expressing cassette are shown. **(B)** The sensitivity and specificity of three primer pairs were determined by PCR in a 20 μL volume containing 50 ng zebrafish genomic DNA as template and transgenic plasmids (0, 1, 5, 10, 20, 50 or 100 copies).

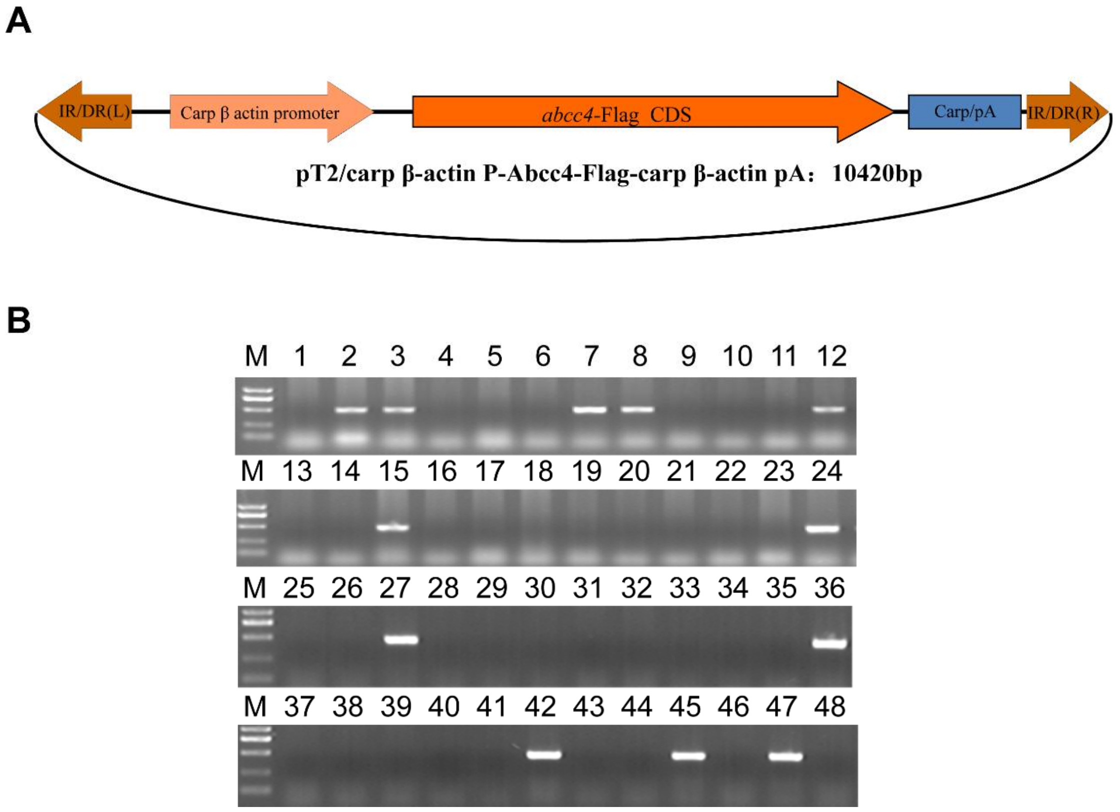

Figure S3 – Screening *abcc4*-transgenic zebrafish in F_1 generation.

(A) Structure of transgenic construct. It includes a strong promoter and a polyadenylation signal sequence from carp β-actin gene, zebrafish *abcc4* coding sequence (CDS) with a0 Flag-tagged at the carboxyl terminus for transgene expression analysis. **(B)** PCR screening of positive F_1 individuals with primer pairs of *abcc4*-F/ *abcc4*-R. M: DNA ladder; lanes 1-48: individual DNA samples. The positive sample gave rise to a 468 bp DNA band.
